# Disparities in the Burden of HIV/AIDS in Canada

**DOI:** 10.1371/journal.pone.0047260

**Published:** 2012-11-27

**Authors:** Robert S. Hogg, Katherine Heath, Viviane D. Lima, Bohdan Nosyk, Steve Kanters, Evan Wood, Thomas Kerr, Julio S. G. Montaner

**Affiliations:** 1 British Columbia Centre for Excellence in HIV/AIDS, Vancouver, Canada; 2 Faculty of Health Sciences, Simon Fraser University, Burnaby, Canada; 3 Faculty of Medicine, University of British Columbia, Vancouver, Canada; University of Pittsburgh, United States of America

## Abstract

**Background:**

We aimed to characterize changes in patterns of new HIV diagnoses, HIV-related mortality, and HAART use in Canada from 1995 to 2008.

**Methods:**

Data on new HIV diagnoses were obtained from Health Canada, HIV-related mortality statistics were obtained from Statistics Canada, and information on the number of people on HAART was obtained from the single antiretroviral distribution site in British Columbia (BC), and the Intercontinental Marketing Services Health for Ontario and Quebec. Trends of new HIV-positive tests were assessed using Spearman rank correlations and the association between the number of individuals on HAART and new HIV diagnoses were estimated using generalized estimating equations (GEE).

**Results:**

A total of 34,502 new HIV diagnoses were observed. Rates of death in BC are higher than those in Ontario and Quebec with the rate being 2.03 versus 1.06 and 1.21 per 100,000 population, respectively. The number of HIV infected individuals on HAART increased from 5,091 in 1996 to 20,481 in 2008 in the three provinces (4 fold increase). BC was the only province with a statistically significant decrease (trend test p<0.0001) in the rate of new HIV diagnoses from 18.05 to 7.94 new diagnoses per 100,000 population. Our analysis showed that for each 10% increment in HAART coverage the rate of new HIV diagnoses decreased by 8% (95% CI: 2.4%, 13.3%)

**Interpretation:**

Except for British Columbia, the number of new HIV diagnoses per year has remained relatively stable across Canada over the study period. The decline in the rate of new HIV diagnoses per year may be in part attributed to the greater expansion of HAART coverage in this province.

## Introduction

Every year 3,300 men and women in Canada are diagnosed with HIV infection. Extrapolating from national estimates, 65,000 Canadians are now living with HIV [1] and based on this 2008 estimate and the current rate of new infections this number could double within the next 15 years.

Canadians living with HIV come from all facets of society and from all regions [1]–[6]. Nearly half of these infections (48%) are among men who have sex with men. Other groups disproportionally affected by HIV in Canada include injection drug users (IDUs), Aboriginal Peoples, and migrants from endemic countries. Though, most Canadians living with HIV reside in Ontario, Quebec and British Columbia, the fastest growing epidemic in Canada is in Saskatchewan where on average 200 new people are diagnosed with HIV infection each year [7].

Untreated HIV infection leads to progressive immune system failure, which in turn leads to the development of opportunistic infections and cancers that ultimately lead to death within 10 to 15 years [8]. Since 1996, with the advent of highly active antiretroviral therapy (HAART) HIV disease has become a chronic manageable condition with a near-normal life expectancy [9]–[11].

Observational research has consistently shown that HAART use is associated with marked reductions in HIV transmission in sero-discordant couples and IDUs [12], [13]. More recently, a prospective randomized trial definitively confirmed that immediate use of HAART decreased genetically linked sexually transmitted HIV infection by 96.3% among HIV sero-discordant couples [14].

Health care in Canada is provided under the auspices of the National Health Insurance Program, often referred to as “Medicare” [15]. This is designed to ensure that all residents have reasonable and affordable access to medically necessary hospital and physician services. Provincial and territorial governments are responsible for the management, organization and delivery of health services for their residents. HAART is subsidized across Canada, however the nature and extent of the subsidy varies across the country. HAART is free in British Columbia, while in Quebec and Ontario it is covered by either public or private insurance through a series of programs, and access may vary according to socioeconomic status [16].

In this study, we aimed to characterize changes in regional patterns for new HIV diagnoses, HAART use, mortality and averted cases in selected Canadian provinces over the past two decades.

## Methods

A population-based approach was used to characterize annual trends new HIV-positive diagnoses, HIV-related mortality, and HAART use in Canada from 1995 to 2008 [17]. Our analysis first focused on all Canada, then the three provinces with the largest epidemic and then the health authorities in British Columbia. Below we describe how these data were collected from published sources (also see Table 1) and how the collected data were then analyzed.

**Table 1:** Data sources used in this study spanning the period 1995 to 2008.

	National	BC, Ontario, and, Quebec	BC only
**New HIV diagnoses**			
Health Canada	x	x	
BC Centre for Disease Control			x
**HIV-related deaths**			
Statistics Canada	x	x	
BC Vital Statistics			x
**Number on HAART**			
IMS* Health		x	
BC Centre for Excellence in HIV/AIDS			x
**Number of HIV+ people**			
Health Canada estimates		x	
Ontario estimates		x	
**Population counts**			
Statistics Canada		x	

* **IMS refers to I**ntercontinental Marketing Services.

### Data sources

The numbers of new HIV-positive cases by province and health authority in British Columbia were obtained from the latest available reports produced by Health Canada [2] and British Columbia Centre for Disease Control [18]. These reports provided the number of new positive tests for each province and territory and for the health authorities in British Columbia.

HIV/AIDS mortality data were obtained from the most recently published reports produced by Statistics Canada [19] and by British Columbia Vital Statistics. Deaths, in which HIV/AIDS was reported as the underlying cause of death, were classified according to the International Classification of Diseases ICD, version 9, from 1987 to 1999 (codes 042–044), [20] or ICD, version 10, from 2000 onwards (codes B20–B24) [21].

The number of people on HAART in British Columbia was obtained from direct counts reported by the single antiretroviral dispensing facility in the province, based at the BC-Centre for Excellence in HIV/AIDS. The number on HAART in Ontario and Quebec were obtained from counts of prescriptions generated by Intercontinental Marketing Services (IMS) Health for these two provinces. These numbers included both retail and non-retail prescriptions. While the retail numbers were readily available, the non-retail values had to be estimated using the number of prescriptions in Canada and market share of non-retail drugs for each province. It should be noted that non-retail accounts for a negligible amount of the prescription in Quebec and Ontario.

Data on the number of HIV-positive people living in British Columbia, Ontario and Quebec were based on estimates produced by Health Canada [1] and by a provincial report for Ontario [22]. Only the 2005 and 2008 values were available for British Columbia and Quebec, the other values were interpolated by taking into account changes in the number of new positive tests per year.

Finally, national and provincial population figures were obtained from annual estimates produced by Statistics Canada [23].

### Outcomes of interest

Five population health indicators were calculated for this study. Rates of new HIV-diagnoses and deaths were calculated for all provinces and for the health authorities in British Columbia. We also calculated the rates of HAART coverage and averted HIV cases and deaths for the three provinces with the largest epidemics – British Columbia, Ontario and Quebec. All rates were expressed per 100,000 populations.

### Statistical analysis

Rates of new HIV-diagnoses and deaths were calculated by dividing the number of cases or deaths by the total population for that province. HIV HAART coverage was estimated by dividing the number of people on HAART in a province by the number of people estimated to be HIV-positive in that jurisdiction. Averted cases and deaths were estimated by using the pre-HAART rate for 1995 and then applying it to subsequent years to estimate the averted cases that were due to either a decrease or increase rates of new cases or deaths in subsequent years. Rates of averted cases and deaths were then calculated by dividing the number of averted cases or deaths by the total population for that province. Trends in new diagnoses were assessed using Spearman rank correlations. 95% Confidence intervals (95% CIs) were calculated for averted HIV cases and deaths.

In order to assess the association between HAART coverage and rate of positive tests, we used generalized estimating equations (GEE) to solve a negative binomial regression. The GEE used an autoregressive correlation matrix to account for the clustering of observations within provinces, across time. An offset accounted for the differences in provincial population sizes and the negative binomial accounted for the over-dispersion. As we do not have reliable data on the distribution of CD4 counts for the whole population infected with HIV in these three provinces, HAART coverage is based on everyone estimated to be infected rather than only those eligible for treatment.

The BC Centre for Excellence in HIV/AIDS and the researchers are funded by the British Columbia Government, as well as by peer-reviewed grants and by foundations or industry grants (see full disclosure statement). The funding sources had no role in the choice of methods, the contents or form of this work, or the decision to submit the results for publication. The Centre's HIV/AIDS Drug Treatment program has received ethical approval from the University of British Columbia Ethics Review Committee at its St. Paul's Hospital site. The program also conforms with the province's Freedom of Information and Protection of Privacy Act.

## Results

In 2008, there were an estimated 65,000 people living with HIV in Canada, with 55,947 (86.1%) residing in the provinces of Ontario, Quebec and British Columbia (see Table 2). A total of 5,141, 8,753, and 6,587 individuals were on HAART in these three provinces, respectively; with highest rate of HAART coverage being in British Columbia at 45%, followed by Quebec at 37% and Ontario at 32% in 2008. If 1995 rates applied Quebec and British Columbia had the highest rates of averted deaths, followed by Ontario.

**Table 2:** Characteristics of those infected with HIV in British Columbia, Ontario and Quebec, 2008.

	British Columbia	Ontario	Quebec
**HIV-positive**			
Mid-point estimate	11,400	26,627	17,920
**HIV-related deaths**			
Number	89	137	94
Rate per 100,000	2.03	1.06	1.21
**HIV diagnoses**			
Number	348	1,121	638
Rate per 100,000	7.94	8.67	8.23
**On HAART**			
Estimated number	5,141	8,753	6,587
Rate per 100,000	117.27	59,61	70.88
HAART coverage (%)	45.10	32.87	36.76
**Averted HIV cases**			
Estimated number	348 (312, 387)	1,121(1056,1189)	638 (589, 689)
Rate per 100,000	7.94 (7.12, 8.83)	8.67 (8.17,9.19)	8.23 (7.60, 8.89)
**Averted HIV-related deaths**			
Estimated number	257(227, 290)	669(619, 722)	532(488, 579)
Rate per 100,000	5.87(5.18, 6.62)	5.17(4.79, 5.58)	6.87(6.30, 7.47)

**Figure 1:**
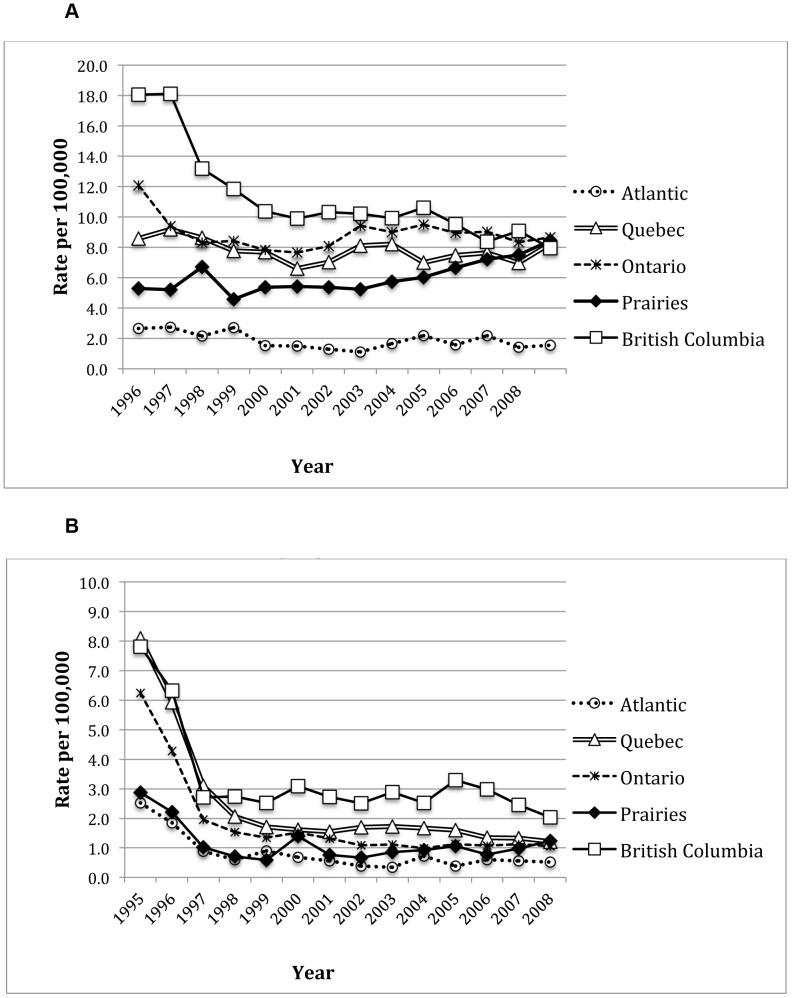
Rates of new HIV-related diagnoses and deaths in Canada. **Panel A**: New HIV-positive cases, by region, 1995–2008. **Panel B**: HIV-related deaths, by region, 1995–2008.

Figure 1 highlights temporal changes in rates of new HIV diagnoses (Panel A) 1995 to 2008 and in rates of HIV-related mortality (Panel B) from 1995 to 2008. A total of 34,502 new HIV diagnoses occurred in Canada from 1995–2008. Over the study period, British Columbia was the only province with a statistically significant decrease in new HIV diagnoses from 18.05 to 7.94 per 100,000 population (p<0.001). New HIV diagnoses per year remained essentially constant for all other provinces except for the Prairies, where rates increased four fold (driven by new infections in the province of Saskatchewan). A total of 8,546 HIV-related deaths were reported by Statistics Canada for the years 1995–2008. Although, rates of mortality decreased in all provinces and regions, the highest rate of decline was in Quebec with 7-fold decrease over the study period. Rates in Ontario and British Columbia also decreased 6 and 4-fold respectively.

**Figure 2:**
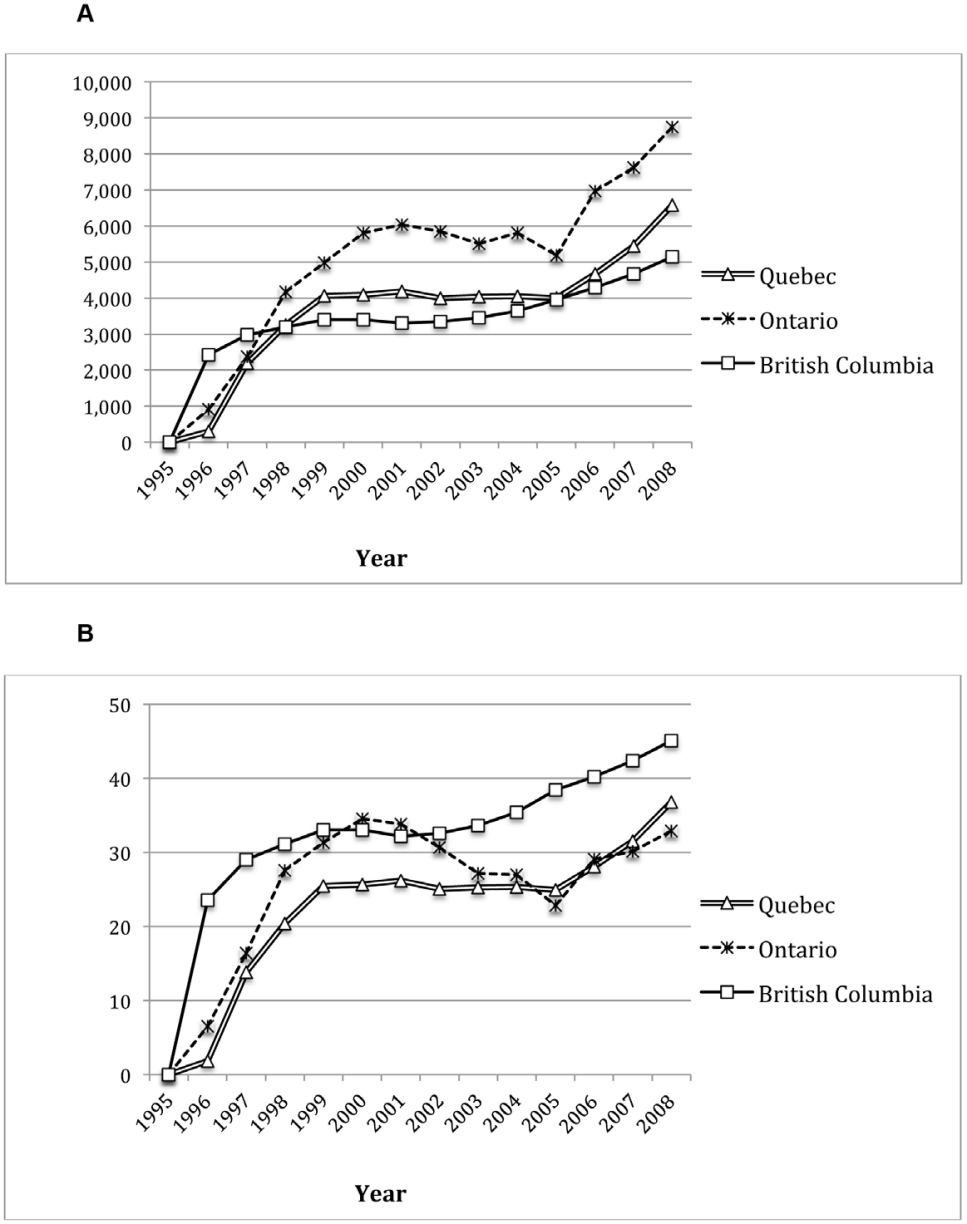
HAART use in British Columbia, Ontario and Quebec, 1995–2008. **Panel A**: Number of HIV infected individuals on HAART. **Panel B**: Percent HAART coverage.

The estimated number of HIV infected individuals on HAART and the rate of HAART coverage for the provinces of Ontario, Quebec and British Columbia, from 1995 to 2008 are shown in Figure 2 (Panels A and B). Based on IMS Health figures for Quebec and Ontario and direct counts for British Columbia, we observed significant increases in HAART use in all three provinces over the study period. Prior to 1996 only a small group of people were on HAART as part of a prospective clinical trial in BC [24]. From 1996 to 2009, the number of HIV infected individuals on HAART increased from 914 to 8,753 in Ontario, 295 to 6,587 in Quebec and 2,419 to 5,625 in British Columbia. HAART coverage was highest in British Columbia, followed then by Quebec and Ontario.

**Figure 3:**
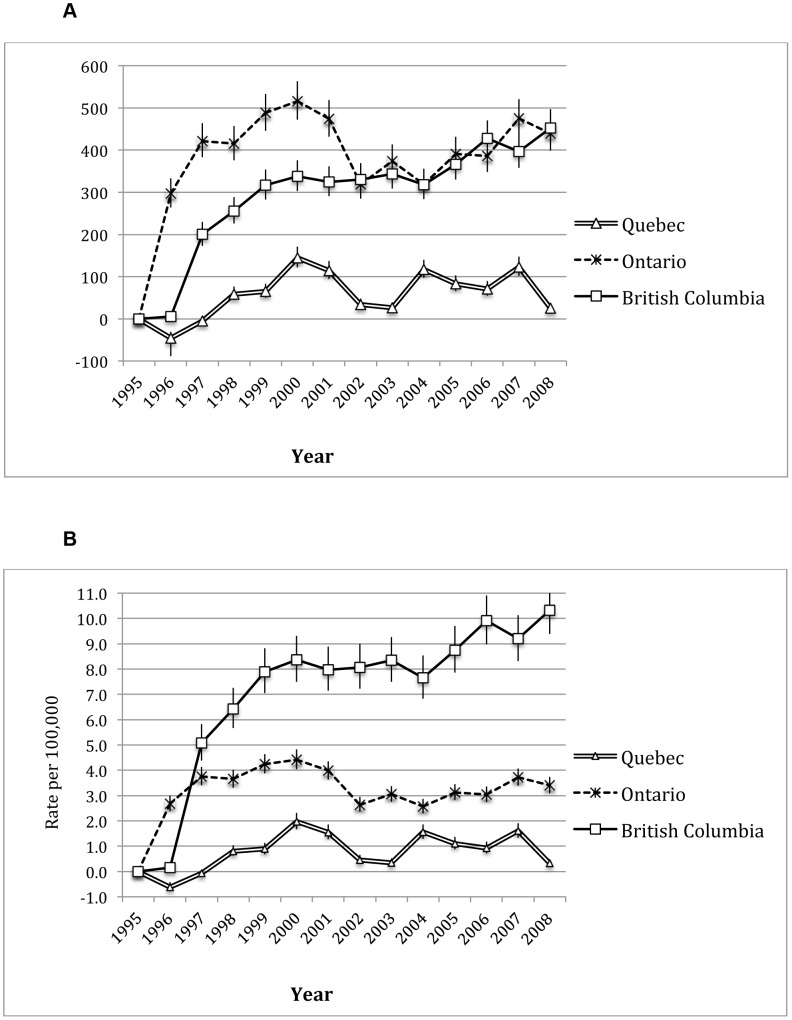
Averted HIV cases in British Columbia, Ontario and Quebec, 1995 to 2008. **Panel A**: Number of averted cases per year with 95% CIs. **Panel B**: The rate of averted cases per year with 95% CIs.

The results of the negative binomial regression showed that the rate of new HIV diagnoses decreased by 8% (95% CI: 2.4%, 13.3%) for each increase of 10% in HAART coverage. Figure 3 (Panels A and B) highlights number and rates of averted HIV cases for the provinces of British Columba, 1995–2008. British Columbia has averted more cases of HIV than Quebec and Ontario combined. In 2008, British Columbia averted 10.33 cases per 100,000 population (95% CI: 9.39, 11.34) compared to 3.40 (95% CI: 3.09, 3.73) and 0.33 (95% CI: 0.22, 0.49) averted cases per 100,0000 population for Ontario and Quebec respectively.

**Figure 4:**
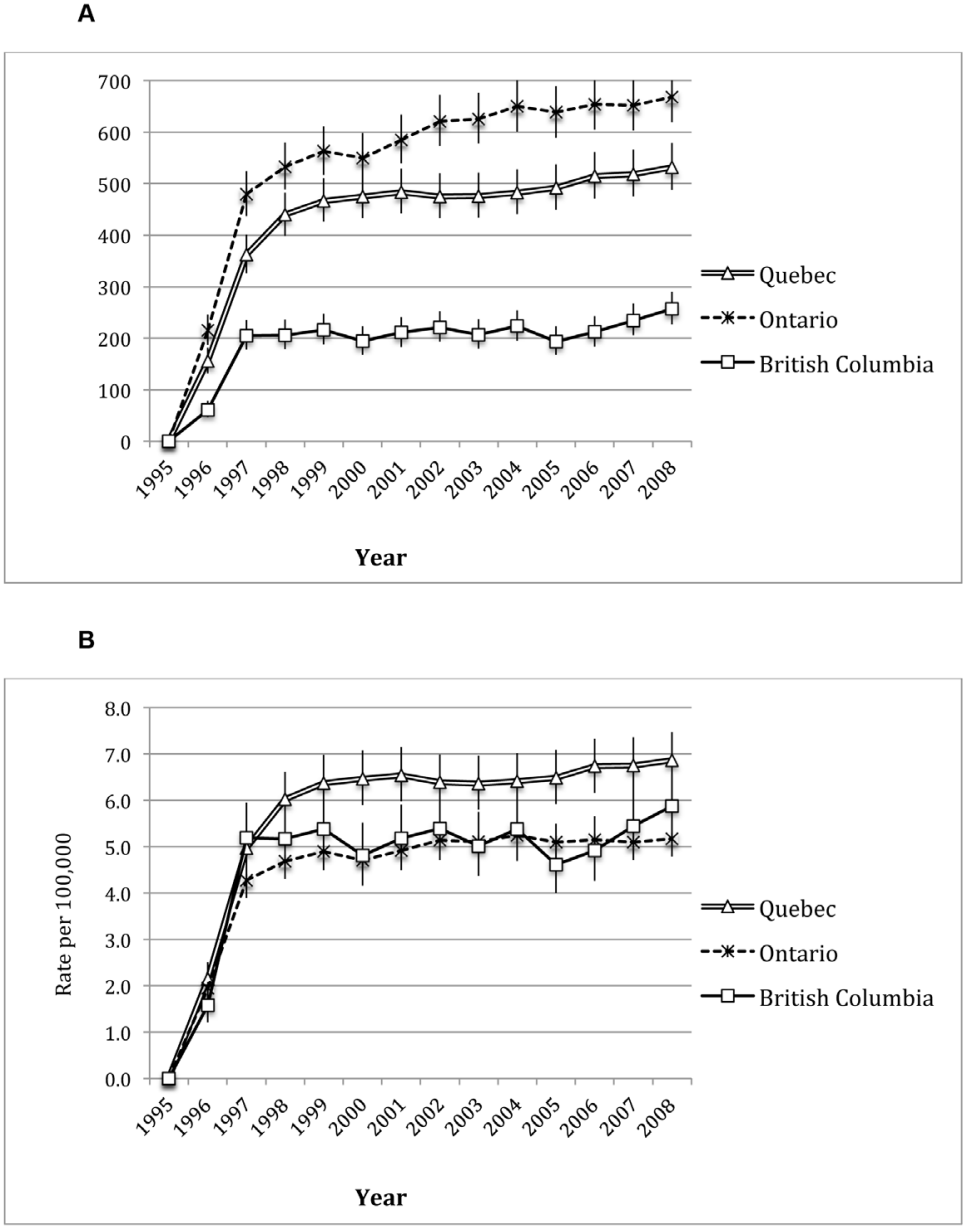
Averted HIV deaths in British Columbia, Ontario and Quebec, 1995–2008. **Panel A**: Number of averted deaths per year with 95% CIs. **Panel B**: The rate of averted deaths per year with 95% CIs.

Figure 4 (Panels A and B) highlights number and rates of averted HIV deaths for the provinces of British Columba, Ontario and Quebec 1995–2008. In 2008, the rate of averted deaths was 5.87 per 100,000 population (95% CI: 5.18, 6.62) in British Columbia compared to 5.17 (95% CI: 4.79, 5.58) and 6.87 (95% CI: 6.30, 7.47) averted deaths per 100,000 population for Ontario and Quebec respectively.

**Figure 5:**
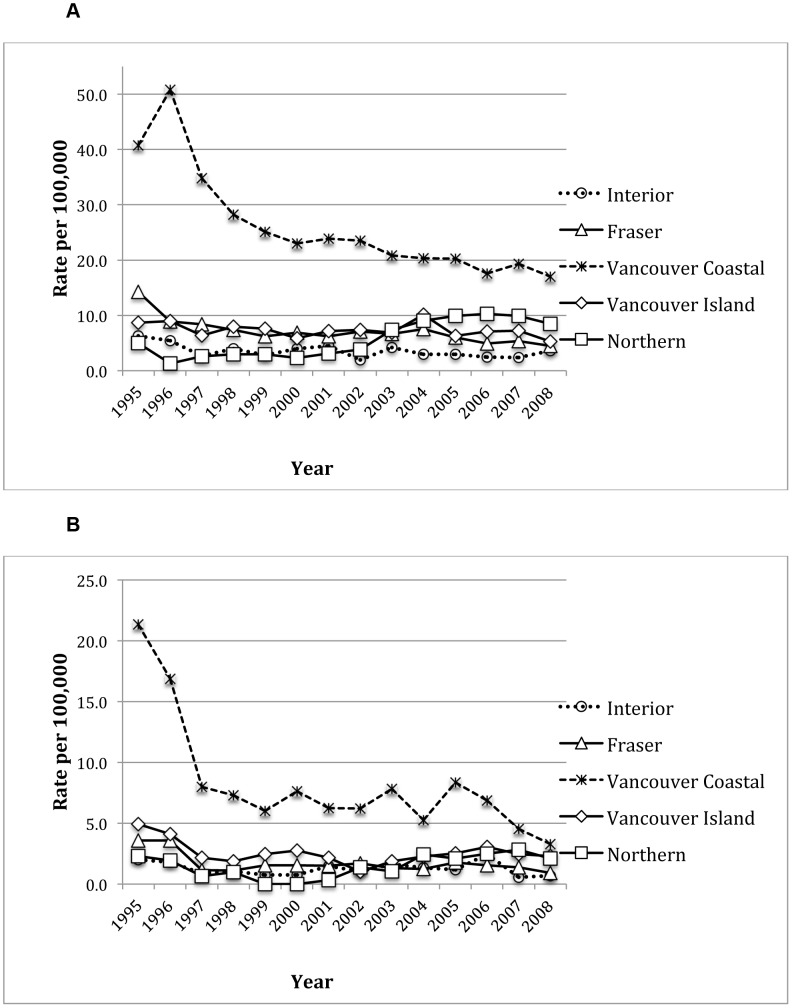
Rates of new HIV-related diagnoses and deaths in BC. **Panel A**: New HIV-positive cases, by health authority, 1995–2008. **Panel B**: HIV-related deaths, by health authority, 1995–2008.

Figure 5 (Panels A and B) highlights rates of new HIV-related diagnoses and deaths in British Columbia for the years 1995 to 2008. The cumulative number of new HIV diagnoses in the province was 6,374 over the study period. Rates of new HIV diagnoses decreased significantly in all health authorities but most notably in the Vancouver Coastal Health Authority. Over the study period, a total of 1,956 HIV-related deaths were observed. The largest decline in rates of new diagnoses and mortality was again observed in the Vancouver Coastal Health Authority.

## Interpretation

Our results demonstrate that for each 10% incremental increase in HAART coverage in the provinces of British Columbia, Ontario, and Quebec, the rate of new HIV diagnoses decreased by 8% (95% CI: 2.4%, 13.3%). British Columbia stands alone in Canada, as the only jurisdiction showing a steady decline in the rate of HIV new diagnoses. These disparities maybe at least partially attributed to the greater expansion of HAART coverage in British Columbia, especially in the Vancouver Coastal Health Authority [25], as well as the implementation of other HIV prevention interventions, including novel harm reduction strategies aimed at IDUs [26], [27].

Rates of mortality have also decrease sharply, particularly in Quebec and to a lesser extent in Ontario. Rates of mortality have declined less in BC. To what extent this is a true phenomenon or is due to an ascertainment bias remains to be established. The latter could be due to enhanced data capture regarding mortality in BC with regular linkages with Vital Statistics enhanced by direct physician reporting to the centralized BC-CfE program.

Since the advent of HAART, HIV-positive individuals accessing treatment have seen a substantial reduction in HIV-related morbidity and mortality [28], [29]. The life expectancy of HIV-positive individuals on therapy now approaches that of uninfected individuals, transitioning HIV from a fatal disease to a manageable chronic condition [11], [30]. Further, a large body of global research suggests that widespread access to HAART plays an important role in reducing HIV incidence at the population level. The concept of “HIV Treatment as Prevention” is founded on the basis that HAART lowers the amount of virus in the body and can thus reduce the chance of treated individuals spreading HIV. HAART has shown to predictably prevent vertical and percutaneous HIV transmission [31], and to curb sexual transmission between sero-discordant couples [12], [13]. Increased HAART access has been associated with reduced HIV incidence by approximately 50% in Taiwan [32], 60% in San Francisco [33] and 60% in British Columbia [17]. Most recently, HPTN 052 – a randomized trial of HIV sero-discordant (primarily heterosexual) couples – provided definitive proof of the efficacy of HIV Treatment as Prevention [14]. The study reported a 96% decrease in the risk of sexual transmission of HIV with immediate HAART in this setting. Of note, immediate HAART was also associated with a 41% decrease in the combined endpoint of disease progression and death, as well as an 83% reduction in the incidence of extra-pulmonary TB. Consequently, it has been argued that rapid expansion of HAART coverage should be considered a clinical and human rights priority as a means to improve HIV-related quality of life and survival, and to reduce HIV incidence and eventually HIV prevalence [25]. Vital statistics reports indicate that HIV/AIDS-related mortality have steadily decreased in Canada since 1995, as a result of the increased access to HAART [19]. However, our results show that HIV/AIDS-related mortality rates were consistently above the national average in some provinces. This relates to the fact that the HIV is not randomly distributed within the population, but rather is overrepresented within certain groups, which are expected to have higher rates of mortality, such as injection drug users and First Nations individuals in British Columbia [2]. Lack of access to health care services and limited uptake of HAART within these populations [34] have previously been shown to be important drivers of excess HIV/AIDS-related mortality in Canada, despite the existence of a socialized medical system nationally [16].

Readers should be cautious when interpreting our results. New HIV-diagnoses are not equivalent to incident infections, as they may have occurred over a variable period of time before the first positive test was obtained. Therefore, true incidence cannot be calculated due to impact of delayed and undiagnosed HIV infections, which Health Canada estimates may reach 26 per cent of cases [35]. HIV/AIDS-related mortality rates are underestimated, as problems of misdiagnosis and underreporting are also common, particularly with respect to the reporting of the underlying causes of death. We have previously shown that physician reporting underestimates HIV/AIDS-related mortality by up to 40% [36] and that a large proportion of HIV-positive men and women on HAART no longer die directly of HIV-related complications [37]. As such, the figures presented in this study may significantly underestimate the impact on HIV/AIDS-related mortality rates. Readers should also note, while the number of individuals on HAART in British Columbia was derived from the single antiretroviral drug repository, the same number for Ontario and Quebec was based on IMS Health data, which may not be equally accurate, as it reflects drug distributed rather than patients on therapy. Finally, estimates of HAART coverage are based on those infected in the province rather those that are eligible for treatment, as we do not have accuracy estimates of CD4 distributions in these provinces over time.

### Conclusions

We demonstrated that for each 10% increase in HAART coverage in three provinces with the largest epidemic the rate of new HIV diagnoses decreased by 8% (95% CI: 2.4%, 13.3%). Except for British Columbia, the number of new HIV diagnoses per year has remained relatively stable across Canada over the study period. We believe this decline in number of new infections may be at least partially attributed to the greater expansion of HAART coverage, which is consistent with the recent data in support of “HIV Treatment as Prevention” [25].
