# Correction: Disparities in the Burden of HIV/AIDS in Canada

**DOI:** 10.1371/journal.pone.0209045

**Published:** 2018-12-06

**Authors:** Robert S. Hogg, Katherine Heath, Viviane D. Lima, Bohdan Nosyk, Steve Kanters, Evan Wood, Thomas Kerr, Julio S. G. Montaner

There is an error in the third sentence of the results subsection of the Abstract. The correct sentence is: The number of HIV-infected individuals on HAART increased from 2,081 in 1996 to 20,431 in 2008 in the three provinces (nearly 10-fold increase).

There is an error in the fourth sentence of the second paragraph of the Results section. The correct sentence is: New HIV diagnoses per year remained essentially constant for all other provinces except for the Prairies, where rates increased two-fold (driven by new infections in the province of Saskatchewan).

There is an error in the penultimate sentence of the third paragraph of the Results section. The correct sentence is: From 1996 to 2009, the number of HIV infected individuals on HAART increased from 914 to 8,753 in Ontario, 295 to 6,587 in Quebec and 872 to 5,091 in British Columbia.

There is an error in the sixth sentence of the third paragraph of the Interpretation section. The correct sentence is: Increased HAART access has been associated with reduced HIV incidence by approximately 50% in Taiwan [32], 60% in San Francisco [33] and 40% in British Columbia [17].

There are errors in Fig 2 and Table 2. Please see the corrected Fig 2 and Table 2 here.

**Fig 2:**
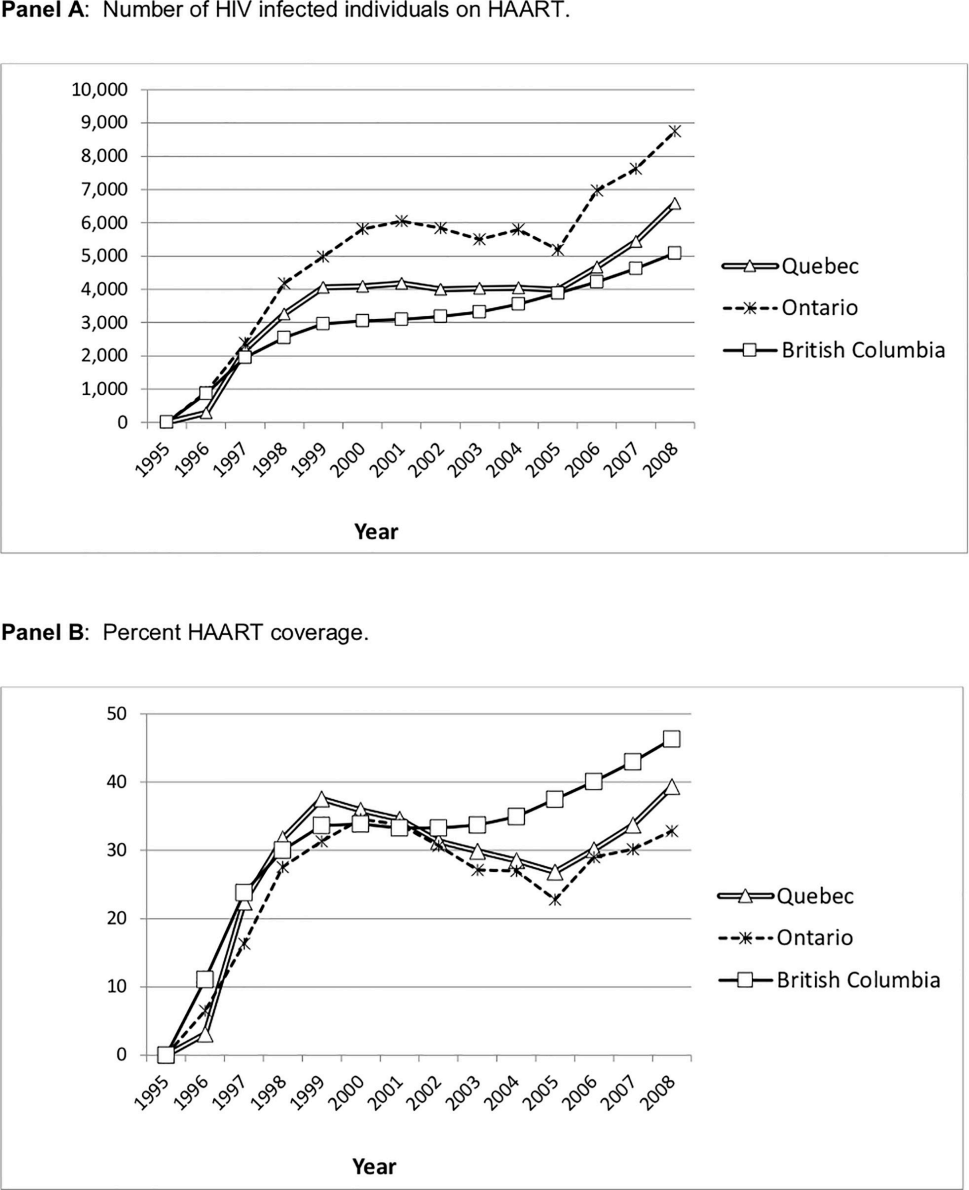
HAART use in British Columbia, Ontario and Quebec, 1995–2008. **Panel A:** Number of HIV infected individuals on HAART. **Panel B:** Percent HAART coverage.

**Table 2:** Characteristics of those infected with HIV in British Columbia, Ontario and Quebec, 2008.

	British Columbia	Ontario	Quebec
**HIV-positive**			
**Mid-point estimate**	**10,992**	**26,627**	**16,744**
**HIV-related deaths**			
**Number**	**89**	**137**	**94**
**Rate per 100,000**	**2.0**	**1.1**	**1.2**
**HIV diagnoses**			
**Number**	**348**	**1,121**	**638**
**Rate per 100,000**	**7.9**	**8.7**	**8.2**
**On HAART**			
**Estimated number**	**5,091**	**8,753**	**6,587**
**Rate per 100,000**	**116.1**	**67.7**	**85.0**
**HAART coverage (%)**	**46.3**	**32.9**	**39.3**
**Averted HIV cases**			
**Estimated number**	**453 (411, 495)**	**439 (398,480)**	**26 (16, 36)**
**Rate per 100,000**	**10.3 (9.4, 11.3)**	**3.4 (3.1, 3.7)**	**0.3 (0.2, 0.5)**
**Averted HIV-related deaths**			
**Estimated number**	**257(227, 290)**	**669(619, 722)**	**532(488, 579)**
**Rate per 100,000**	**5.9 (5.2, 6.6)**	**5.2 (4.8, 5.6)**	**6.9 (6.3, 7.5)**

## References

[1] Hogg RS, Heath K, Lima VD, Nosyk B, Kanters S, Wood E, et al (2012) Disparities in the Burden of HIV/AIDS in Canada. PLoS ONE 7(11): e47260 10.1371/journal.pone.0047260 23209549 PMC3507870

